# Structure–Performance Relationship of Anionic Polyacrylamide in Pyrite-Containing Tailings: Insights into Flocculation Efficiency

**DOI:** 10.3390/polym17081055

**Published:** 2025-04-14

**Authors:** Steven Nieto, Eder Piceros, Gonzalo R. Quezada, Pedro Robles, Ricardo I. Jeldres

**Affiliations:** 1 Advanced Mining Technology Center (AMTC), Universidad de Antofagasta, Antofagasta 1240000, Chile; yeison.nieto.mejia@ua.cl; 2 Faculty of Engineering and Architecture, Universidad Arturo Prat, P.O. Box 121, Iquique 1100000, Chile; edpicero@unap.cl; 3 Escuela de Ingeniería Civil Química, Universidad del Bío-Bío, Concepción 4030000, Chile; grquezada@ubiobio.cl; 4 Escuela de Ingeniería Química, Pontificia Universidad Católica de Valparaíso, Valparaíso 2340000, Chile; 5 Departamento de Ingeniería Química y Procesos de Minerales, Facultad de Ingeniería, Universidad de Antofagasta, Antofagasta 1240000, Chile

**Keywords:** pyrite flocculation, anionic polyacrylamide, aggregate structure

## Abstract

Functional polymeric materials play a critical role in optimizing flocculation and sedimentation processes in mining tailings, where complex interactions with mineral surfaces govern polymer performance. This study examines the structure–performance relationship, which describes how the internal structure of aggregates (e.g., compactness, porosity and fractal dimension) influences sedimentation behavior, specifically for anionic polyacrylamide (SNF 704) in kaolin-quartz-pyrite suspensions at a pH of 10.5. Using focused beam reflectance measurement (FBRM) and static sedimentation tests, we demonstrate that pyrite exhibits the highest flocculant adsorption capacity, inducing a train-like polymer conformation on its surface. This reduces the formation of effective polymeric bridges, resulting in less compact and more porous aggregates that negatively impact sedimentation rates. Increasing the flocculant dosage improves the capture of fine particles; however, at high pyrite concentrations, rapid saturation of adsorption sites limits flocculation efficiency. Additionally, the fractal dimension of the aggregates decreases with increasing pyrite content, revealing more open structures that hinder consolidation. These findings underscore the importance of optimizing polymer dosage and tailoring flocculant design to the mineralogical composition, thereby enhancing water recovery and sustainability in mining operations. This study highlights the role of structure–property relationships in polymeric flocculants and their potential for next-generation tailings management solutions.

## 1. Introduction

Pyrite (FeS_2_) is a common mineral in copper deposits, and its efficient separation is essential to avoid contamination of mineral concentrates in flotation processes. Traditionally, pyrite depression is carried out by controlling the pH, bringing it to values above 10 with depressing reagents such as lime, which makes pyrite hydrophilic and facilitates its separation from valuable minerals [1,2].

Subsequently, the flotation tailings are concentrated in the thickening stages, where the mineralogy of the tailings plays a crucial role in determining the operation’s efficiency. There is a substantial body of scientific literature that addresses this problem, with a particular focus on the impact of clays, which are lightweight minerals with a specific gravity of approximately 2.5 g/cm^3^, irregular morphology and charge densities that vary on their edges and faces [3,4,5,6]. There are various types of clays, such as those from the kaolin group or the smectite group, which have different properties. For example, the latter swells in contact with water. These characteristics present considerable challenges during the thickening stages, as they reduce sedimentation rates and compaction of the thickened tailings discharged into the thickener underflow. Nieto et al. [7] investigated the impact of two types of water (industrial and seawater) on flocculation, sedimentation rate and compressive yield strength in kaolin suspensions at a pH of 7. They also analyzed the relationships between these properties and the flocculant dose, aggregate size and fractal dimension. One of their most significant findings was that the presence of salt and low doses of SNF 704 flocculant enhanced the consolidation of kaolin pulps under compression, favoring the formation of face-to-face structures within the kaolin. However, under high-salinity conditions, the flocculant’s capacity to form polymeric bridges with the particle surfaces is reduced, resulting in a greater number of smaller aggregates with more compact structures.

However, there are no studies investigating the impact of pyrite on the operational performance. It must be considered that pyrite, due to its high density ($\approx 5\;g/{cm}^{3}$), tends to sediment faster than other minerals present in the tailings, such as quartz and clays. However, the efficiency of the process also depends on the interaction of the minerals with the flocculants added to the feedwell of the thickeners and the efficiency in the management of these reagents [8,9], which usually correspond to high molecular weight soluble polymers, such as anionic polyacrylamide, which have functional groups such as carboxylate. The anionic carboxylate functionality (COO^−^) favors the chain extension in solution. Also, it helps adhere to the surface with loops mode [10], mainly through ionic bridges [11], using salts from industrial waters. Pyrite is susceptible to oxidation under certain conditions, which could generate sulfuric acid in the medium, affecting the pH and rheological properties of the mud [12]. Fuerstenau et al. [13] and López Valdivieso et al. [14] identify isoelectric points between pH 4.5 and 6.4 associated with pyrite oxidation in this pH range. In the final oxidation stage, surface products such as ferric oxyhydroxides (e.g., goethite) are generated, which partially cover the pyrite and are responsible for pyrite’s positive zeta potential in the pH range. Paredes et al. [15] report that the presence of positive charges of monovalent ions such as Cs^+^, K^+^, Na^+^, and Li^+^ are adsorbed on the anionic sites of the pyrite surface, reducing the value of the negative zeta potential. They also indicate that a higher concentration of cations generates a more pronounced decrease in the absolute zeta potential.

The most recent studies evaluating particle flocculation consider microscopic analysis to directly measure properties of particle agglomerates, such as size distribution [16,17,18,19,20], strength [21,22] or fractal dimension [23,24,25]. This last parameter refers to the structure of the aggregates and has values between one and three. As the fractal dimension increases, the floc is more compact, increasing yield stress values and sedimentation velocity [7,26,27]. Several studies have examined the structure of suspended aggregates by quantifying the fractal dimension under different hydrodynamic conditions, dosing strategies, mineralogy and flocculation time [6,28,29,30]. For example, Leiva et al. [31] employed PAM SNF 704 to investigate the effect of kaolin on the structure of particle aggregates in freshwater at pH 7.5. The results showed that the fractal dimension decreased with increasing kaolin content, resulting in irregular and porous aggregates that can reduce the sedimentation rate and solids consolidation. In operational terms, a higher kaolin content in the suspension can negatively impact the efficiency of solid–liquid separation, increase the volume of sludge in tailings deposits and potentially lead to a higher demand for flocculants to enhance particle aggregation.

Several investigations have examined the impact of fines and clay minerals on the flocculation and settling of HPAM-treated tailings under both low and high alkalinity conditions [9,31,32]. However, the effect of pyrite on the flocculation efficiency of clayey tailings is unknown. Although pyrite is frequently present in tailings and exhibits distinct surface chemistry and density compared to clays and quartz, its influence on polymer performance and floc structure has not been systematically evaluated.

This represents a significant gap in the current understanding of tailings treatment, particularly under alkaline and saline conditions that are typical of modern mining operations using seawater or recycled water. In this context, this research aims to evaluate the impact of pyrite on the structure and flocculation efficiency of clayey tailings treated with high-molecular-weight polyacrylamide (HPAM) under high alkalinity conditions (pH 10.5). By analyzing the changes in the fractal dimension of the aggregates, the quality of the supernatant, and the sedimentation, the use of HPAM in systems with high pyrite concentration is sought to be optimized, contributing to improved water recovery and the sustainability of mining processes.

## 2. Materials and Methods

### 2.1. Materials

The solution to prepare the kaolin-quartz-pyrite (KQP) suspensions consisted of industrial water with a concentration of 0.01 M NaCl and 0.005 M CaCl_2_. The process used distilled water, analytical-grade reagents and lime as a pH modifier.

**Figure 1:**
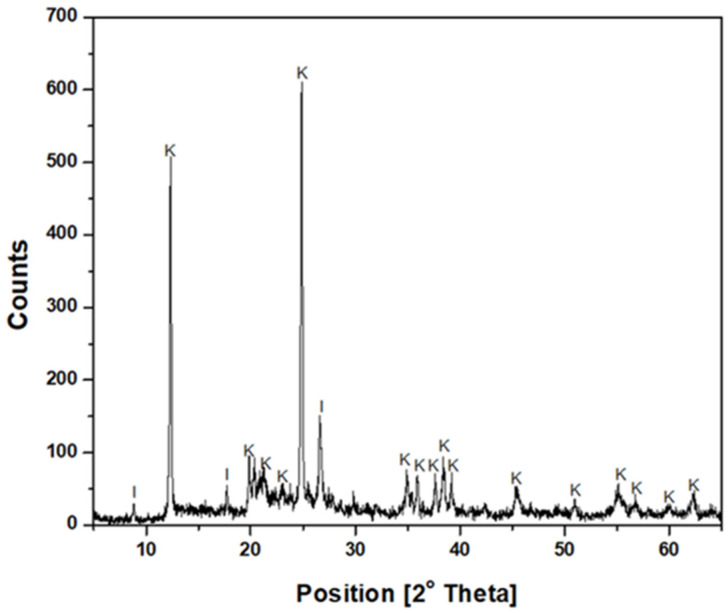
X-ray diffractogram of kaolin (K: Kaolinite, I: Illite).

**Figure 2:**
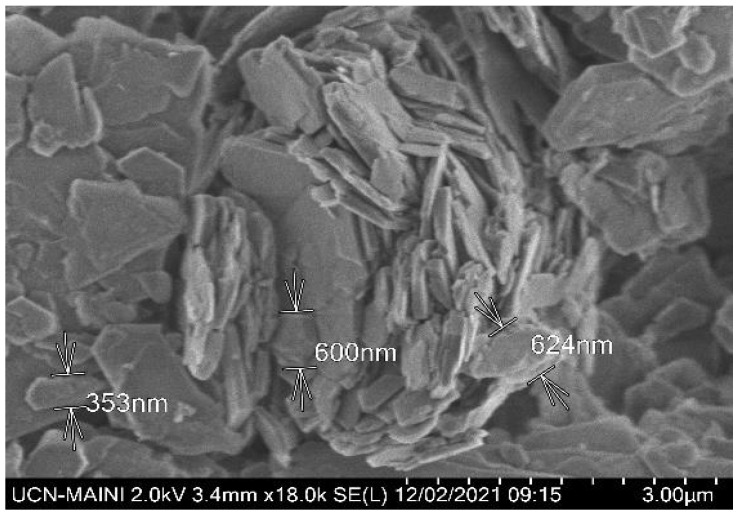
High-resolution SEM image showing kaolin morphology.

The kaolin particles were purchased from Sigma-Aldrich (St. Louis, MO, USA). The mineralogical composition was determined using an advanced Bruker D8 X-ray diffractometer, whose diffractogram (Figure 1) confirmed the predominant presence of kaolinite and illite. In addition, the SEM micrograph (Figure 2) revealed a submicron population of kaolinite particles (<1 µm) with a hexagonal lamellar crystalline structure, undetectable by the focused beam reflectance measurement (FBRM) probe.

TOPAS (total pattern analysis solution) software version 6 was used to quantify quartz using the XRD technique. Quartz particles were obtained from Donde Capo (Santiago, Chile), with a SiO_2_ content greater than 99% by weight. Pyrite particles were purchased from Ward’s Science (Rochester, New York, NY, USA). Pyrite samples were ground in a mortar, pulverized and sieved to obtain a particle size distribution ranging from −75 µm to +38 µm. The kaolin, quartz, and pyrite minerals had densities of 2.57, 2.65, and 5.01 kg/m^3^, respectively.

**Figure 3:**
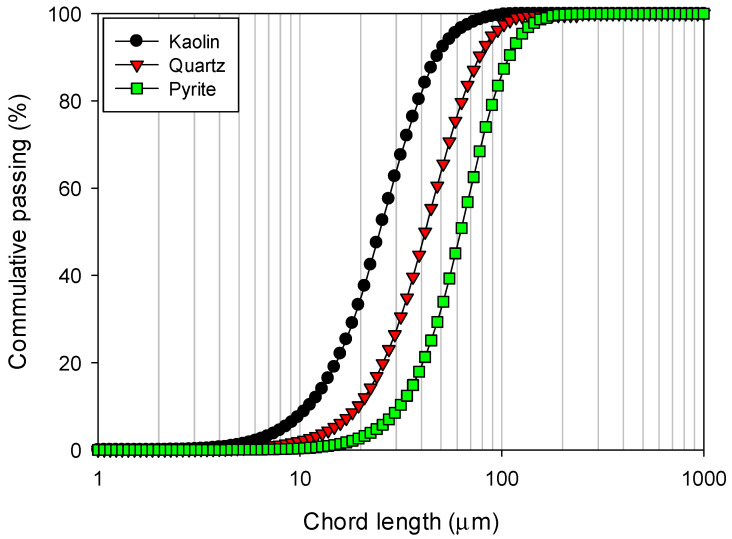
Particle size distributions of kaolin, quartz and pyrite in industrial water, as determined by focused beam reflectance measurement using Particle Track E25 from Mettler–Toledo.

The chord length distributions for kaolin, quartz and pyrite particles were obtained using focused beam reflectance measurement (FBRM) with a Particle Track E25 probe (Mettler Toledo, Columbus, OH, USA) and are presented in Figure 3. These measurements were performed in industrial water to replicate the ionic environment present in actual flocculation tests and to assess particle dispersion and aggregation behavior under realistic conditions. It is observed that kaolin particles show a significantly narrower size distribution compared to pyrite particles, which exhibit the broadest distribution. In terms of percentiles, the analysis revealed that 10% of the particles had sizes below d10 = 11.2 µm for kaolin, 19.9 µm for quartz and 33.2 µm for pyrite.

The flocculant used in the experiments was SNF 704, an anionic polyacrylamide polymer provided by SNF Chile S.A., with a molecular weight of 18 × 10^6^ g/mol and an ionic charge density of 30 to 50% in its anionic functionalities due to partial substitution of acrylamide monomers by acrylate groups (-COO^−^). This substitution yields a highly charged, water-soluble polyelectrolyte that assumes extended conformations in solution at alkaline pH. For its preparation, a mother solution was initially prepared at a concentration of 1 g/L, with constant stirring maintained for 24 h to ensure complete dissolution and stability. An aliquot was taken from this mother solution and diluted to achieve a final concentration of 0.1 g/L, which was used in the experiments. The flocculant doses were expressed as grams of polymer per ton of dry solid (g/t), facilitating direct comparison with industrial dosing parameters.

### 2.2. Flocculant Adsorption

For the adsorption tests, 100 mL suspensions with 2% solids by weight were prepared using industrial water for each of the minerals (kaolin, quartz and pyrite), with the pH adjusted to 10.5. The suspensions were stirred for 4 min at 200 rpm to ensure a homogeneous distribution of the particles. Subsequently, the flocculant SNF 704 was added, and the stirring was maintained for one additional minute at the same speed to promote adsorption without causing mechanical damage to the flocs due to shearing. All experiments were conducted under controlled conditions at a temperature of 20 °C.

After stirring, the mixtures were centrifuged at 2400 rpm for 1 h to separate the mineral particles from the supernatant. The supernatant, containing unadsorbed SNF 704 flocculant, was carefully filtered to avoid particulate contamination, stored in vials and analyzed for total organic carbon (TOC). This analysis was performed using a SHIMADZU brand TOC analyzer, ensuring accurate quantification of the unadsorbed flocculant fraction.

### 2.3. Flocculation–Sedimentation Tests

Synthetic tailings suspensions at 10% solids concentration were prepared using industrial water as the medium. The proportion of kaolin was kept constant at 10%, while the pyrite concentration was adjusted between 3% and 10% and the mixture was completed with quartz. The suspensions were mixed vigorously for 10 min at 500 rpm using a 30 mm diameter polytetrafluoroethylene (PTFE) turbine agitator, placed axially inside a 100 mm diameter and 1 L capacity flocculation vessel. The agitator was positioned at a height of 20 ± 1 mm above the bottom of the vessel. The mixing speed was then reduced to 200 rpm, and the flocculant SNF 704 was added at doses of 10 and 30 g/t. The suspensions thus prepared were used for batch sedimentation tests and characterization of aggregates. In batch sedimentation tests, after 30 s of mixing between the suspension and the flocculant, the suspensions were poured into removable cylindrical vessels with a capacity of 300 cm^3^ and an internal diameter of 35 mm. The vessels were manually inverted four times to homogenize the mixture before proceeding to video recording, which was used to measure the sedimentation rate.

An FBRM (focused beam reflectance measurement) probe was used to characterize the aggregates [16,18,19,20]. The probe was introduced vertically into the suspension, 10 mm above the PTFE stirrer and 20 mm from the central axis. A Mettler Toledo Particle Track G400 equipment was equipped with FBRM technology. The probe generated a focused laser that scanned a circular path at a tangential speed of 2 m/s through a sapphire window. Upon intercepting a particle, the laser emitted a backscatter signal proportional to the particle’s chord length, allowing real-time monitoring of changes in particle size and number in the suspension. These data allowed the evaluation of floc formation and evolution dynamics under different experimental conditions.

### 2.4. Fractal Dimension

The fractal dimension (*D_f_*) is a fundamental tool for characterizing the structure and complexity of the aggregates formed during the thickening process. This parameter can take values between 1 and 3, where 1 represents a one-dimensional linear configuration, while 3 corresponds to a completely solid sphere.

Aggregates with a high fractal dimension are usually compact, dense and more resistant to fragmentation under shear conditions. In contrast, aggregates with a low fractal dimension tend to be open and porous, making them more susceptible to fragmentation and less efficient in sedimentation and consolidation. This parameter is crucial for understanding the dynamics of flocs under different operating conditions and optimizing the dosage of flocculants and the mixing conditions in thickeners.

The Equation (1) proposed by Heath et al. [23] was used to determine the fractal dimension of the aggregates as follows:

(1) $${\;U}_{h}=\frac{d_{agg}^{2}g(\rho_{s}{-\rho }_{l}){\left(\frac{d_{agg}}{d_{p}}\right)}^{D_{f}-3}}{18µ}\;{\left(1-\phi_{s}{\left(\frac{d_{agg}}{d_{p}}\right)}^{3-D_{f}}\right)}^{4.65}$$

where $U_{h}$ is the hindered settling rate in m/s, $\rho_{s}$ and $\rho_{l}$ are the densities of the solid and liquid phases, respectively, and $d_{p}$ and $d_{agg}$ are the average particle size, respectively. Considering the average aggregate size after the flocculation time, µ is the viscosity of the fluid, $\phi_{s}$ is the volume fraction of the solid and $D_{f}$ is the fractal dimension. The hindered settling rate from Section 2.3 was used to determine the fractal dimension, and the squared-weighted chord length distribution obtained with FBRM was used to determine the average aggregate size. The values for each parameter are presented in Table 1.

### 2.5. Zeta Potential Characterization

Suspensions of quartz, kaolin and pyrite with a solid concentration of 1% by weight were prepared using industrial water as a medium. The suspensions were analyzed in the absence and presence of the flocculant SNF 704 at 1 ppm and 5 ppm. The pH of the suspensions was adjusted to 10.5 using lime as a modifier, and the samples were homogenized entirely before proceeding to the measurements.

The zeta potentials of the particles were determined using Litesizer 500 equipment, based on the principle of electrophoretic light scattering using the CmPALS (continuously monitored phase analysis light scattering) technique. The measurements were performed using an Omega Zeta cell, designed to ensure precision in colloidal systems. The experimental conditions were controlled with a temperature of 20 °C and an applied voltage of 220 V. These measurements allowed us to evaluate the impact of the flocculant and the surface chemical interactions of the particles under alkaline conditions typical of flocculation processes in mining.

## 3. Results and Analysis

### 3.1. Sedimentation

In the thickening operation, the initial settling rate is a key parameter for evaluating the efficiency of processes, as it is influenced by variables such as flocculant dosage, the proportion and concentration of solids, pH and water quality. This parameter has a direct relationship with water recovery, as more effective control of solids sedimentation enables the optimization of thickening, achieving a higher concentration of solids in the sediment and releasing a significant amount of water for reuse in upstream operations. This approach not only reduces operating costs but also enhances the sustainability of the mining system.

**Figure 4:**
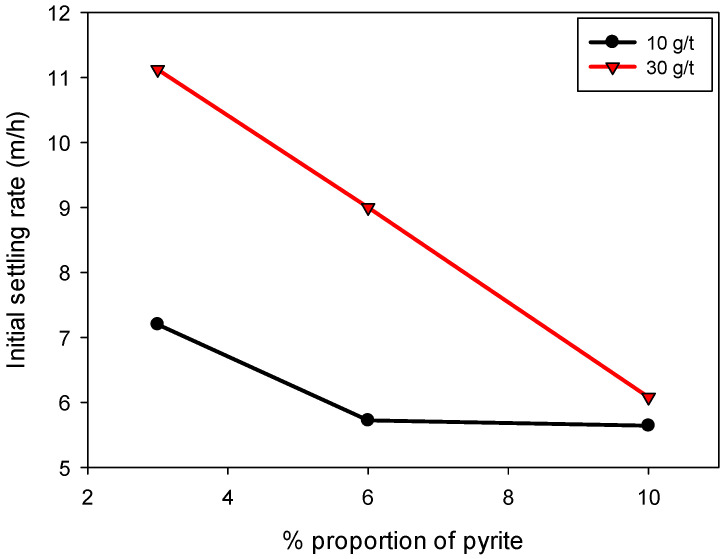
Effect of pyrite solids proportion on the sedimentation rate of kaolin-quartz-pyrite suspensions in industrial water. The mixing speed is 200 rpm, the solid concentration is 10% by weight, the flocculation time is 30 s and the pH is 10.5.

Figure 4 shows that the sedimentation rate increases with the increase in flocculant dosage. This increase is attributed to the formation of polymeric bridges between the particles, facilitated by the flocculant, which leads to the generation of larger aggregates and mass, thus accelerating the sedimentation process. The flocculant used, SNF 704, is particularly effective in forming interlocking particulate networks, which improves sedimentation efficiency [6,33]. The results show that the highest sedimentation rates were achieved in systems with lower pyrite content. For a 30 g/t dosage, the sedimentation velocities ranged from 6.0 to 11.1 m/h, while for a 10 g/t dosage, the values were reduced to a range of 5.6 to 7.2 m/h. These data suggest that, although a higher flocculant dosage promotes faster sedimentation, its effectiveness decreases in the presence of high proportions of pyrite. This result is exciting and counterintuitive since pyrite particles have the largest sizes within the tailings and, simultaneously, the highest specific gravity. These two characteristics significantly favor sedimentation rates. However, another important aspect is the interaction between the particle surface and the flocculant, as well as the agglomeration mechanisms, which are favored in cases with lower pyrite content. This behavior illustrates that floc structure, not just particle size or density, is a key determinant of sedimentation efficiency. The presence of pyrite promotes the formation of larger, but more porous, flocs, which reduces their effective settling velocity despite the high specific gravity of the pyrite particles.

### 3.2. Flocculation Kinetic

**Figure 5:**
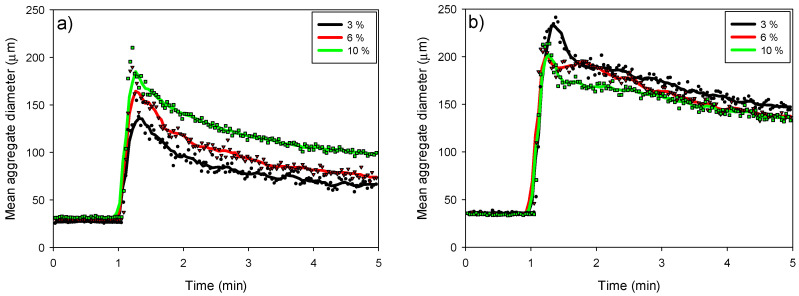
Effect of pyrite percentage on the evolution of the average aggregate size of kaolin-quartz-pyrite suspensions considering flocculant doses of 10 g/t (**a**) and 30 g/t (**b**). The mixing speed is 200 rpm and the solid concentration is 10% by weight with a pH of 10.5.

In this section, the kinetic profiles obtained using the FBRM probe are analyzed, enabling us to study the kinetics of aggregation, restructuring and fragmentation of aggregates in flocculated suspensions of kaolin, quartz and pyrite. The two doses of SNF 704 flocculant used were 10 g/t (Figure 5a) and 30 g/t (Figure 5b). In both cases, after adding the flocculant, the size of the aggregates increased rapidly, reaching a maximum value a few seconds after the start of flocculation. The flocculant dose and the percentage of pyrite in the suspension influenced this maximum size.

With a dose of 10 g/t, the maximum size of the aggregates ranged between 135 and 210 µm, depending on the pyrite proportions, which varied between 3% and 10%, respectively. In contrast, larger aggregates were observed when the dosage was increased to 30 g/t, with sizes ranging from 210 to 250 µm, corresponding to pyrite proportions of 10% to 3%, respectively. This behavior indicates an inverse trend in the effect of pyrite content on aggregate size at both dosages; while at 10 g/t, a higher proportion of pyrite produces larger aggregates, at 30 g/t, a higher proportion of pyrite results in smaller aggregates.

This result is significant to highlight, as the effect of pyrite on the size of the agglomerates differs from the effect it has on the sedimentation rate. This directly suggests that the presence of pyrite causes the flocs to be less compact, resulting in slower sedimentation. Then, at low flocculant doses, larger flocs are generated when the pyrite content increases. The flocculant has a very high affinity for the pyrite surface; therefore, it is expected that the flocculation kinetics would be favored with this mineral. However, this likely leads to a rapid reduction of the available sites on the pyrite for the adsorption of the flocculant, which probably takes a ‘train’ conformation on the surface. This leads to the fact that when the reagent dosage is increased, the growth of flocs is less efficient since the pyrite is closer to its saturation zone. This inverse effect at higher dosages emphasizes that the internal structure of the flocs, influenced by the adsorption conformation of the polymer, plays a more dominant role than the absolute floc size in determining sedimentation performance.

### 3.3. Chord Length Distributions (CLD)

**Figure 6:**
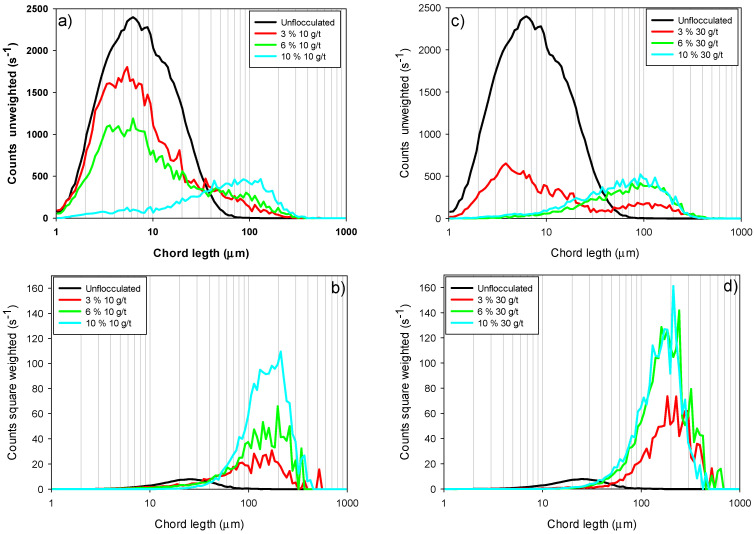
(**a**) Unweighted chord length distribution (CLD) for KQP under a dose of 10 g/t, (**b**) square-weighted CLD for KQP under a dose of 10 g/t, (**c**) unweighted CLD for KQP under a dose of 30 g/t and (**d**) square-weighted CLD for KQP under a dose of 30 g/t. The mixing rate is 200 rpm, the solid concentration is 10 wt%, the flocculation time is 30 s and the pH is 10.5. KQP = kaolin-quartz-pyrite.

Chord length is a fundamental metric for characterizing the size of aggregates or particles in suspensions, whether flocculated or non-flocculated. Figure 6 shows the chord length distributions in two modalities: unweighted (Figure 6a,c) and square weighted (Figure 6b,d), for tailings composed of kaolin-quartz-pyrite (KQP) with different proportions of pyrite in suspension (3–10%). The unweighted distributions allow us to evaluate the temporal evolution of the growth and fragmentation of the smallest aggregates, while the square-weighted distributions emphasize the behavior of the largest aggregates, providing relevant information on the global structure of the system in agreement with what has been reported in the literature [19].

Figure 6a,c show that increasing the proportion of pyrite in the suspension (from 3 to 10%) significantly reduces the amount of non-flocculated fine particles (2–9 µm). This effect suggests that pyrite favors the flocculation of fine particles, thereby improving the process efficiency for both evaluated flocculant doses (10 and 30 g/t). For example, at a dose of 10 g/t, the suspension with 3% pyrite presented a higher fine particle count, reaching a maximum peak of 1784 s^−1^, which decreased to 70 s^−1^ when the proportion of pyrite increased to 10%. Similarly, for a dose of 30 g/t, the maximum fine particle count peak was 646 s^−1^ at 3% pyrite, decreasing to values close to 0 s^−1^ in the fine particle region as the pyrite proportion reached 6% and 10%.

Increasing flocculant dosage also significantly impacted fine particle capture. Maintaining a constant pyrite proportion of 3%, a 30 g/t flocculant dosage fails to capture fine particles completely, with a peak of 646 s^−1^ being recorded. However, this dosage represents a considerable reduction compared to a dosage of 10 g/t, where the peak height reached 1780 s^−1^, indicating an improvement in flocculation efficiency with increasing flocculant.

Furthermore, the size of coarse aggregates increased with flocculant dosage, as evidenced by the squared-weighted chord length distributions. However, opposite trends were observed depending on the dosage applied. For 10 g/t (Figure 6b), the size of coarse aggregates increased with increasing pyrite proportion, reflected by a shift of the distributions to the right. In contrast, at 30 g/t (Figure 6d), the size of coarse aggregates increased as the proportion of pyrite decreased. These results are consistent with those presented in Section 3.2, where the high affinity between pyrite and the flocculant SNF704 is highlighted, favoring significant floc growth. However, at high doses, flocculant oversaturation limits this growth due to the formation of trains, restricting effective particle aggregation. These observations confirm that pyrite enhances the initial capture of fines due to its high affinity for the flocculant. However, the flocs formed are less dense and more fragile at high pyrite content, revealing that aggregate structure—not just size distribution—governs the settling performance under these conditions.

### 3.4. Fractal Dimension

**Figure 7:**
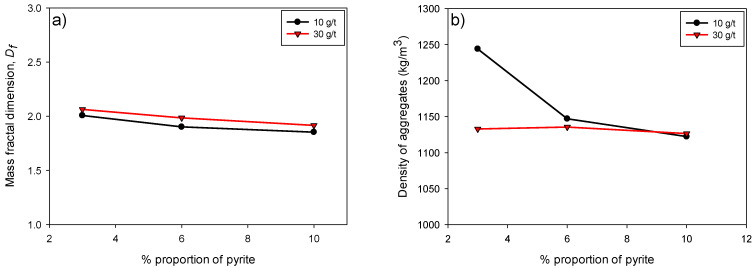
Fractal dimension (**a**) and aggregate density (**b**) as a function of pyrite proportion in flocculated kaolin-quartz-pyrite suspensions at different flocculant doses (10 and 30 g/t). Mixing speed: 200 rpm, solids concentration: 10 wt% and pH: 10.5.

Figure 7a illustrates the variation in fractal dimension (*D_f_*) as a function of the pyrite percentage (3–10%) in flocculated kaolin-quartz-pyrite suspensions for two flocculant doses, 10 and 30 g/t. It can be observed that, for both doses, *D_f_* decreases slightly as the proportion of pyrite in the suspension increases. Thus, the aggregates formed would be less compact and more porous as the pyrite content increases.

Figure 7b shows the variation in the density of KQP tailings aggregates as a function of the proportion of pyrite in suspension. Lower density values were presented in the presence of a higher flocculant dosage, the difference being mitigated as the proportion of pyrite increased. Several studies have shown that larger aggregates, or macroaggregates, generally contain more trapped fluid than smaller aggregates, which significantly reduces their density, leaving it barely higher than the surrounding fluid [34,35]. This trend further supports the structure–performance relationship proposed, where a lower fractal dimension (i.e., a more open structure) correlates with lower sedimentation efficiency, regardless of floc size or density.

### 3.5. Minerals Surface Adsorption

**Figure 8:**
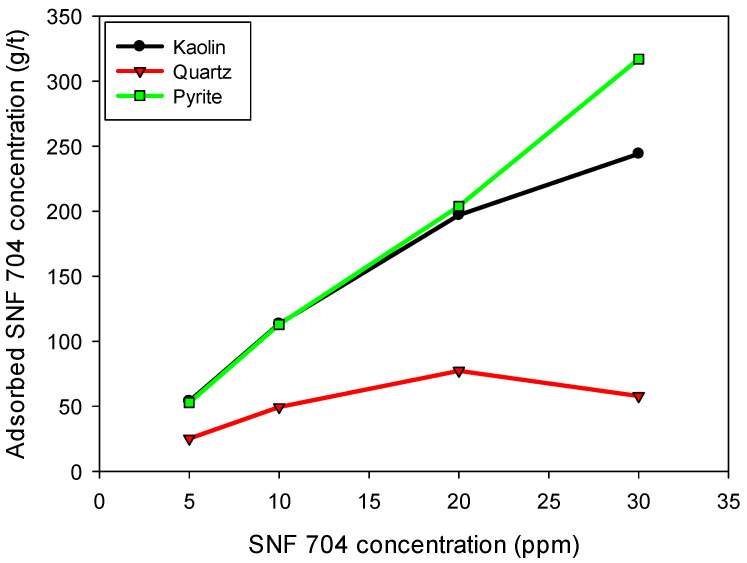
Adsorption of SNF 704 flocculant on kaolin, quartz and pyrite surfaces in the presence of industrial water at pH 10.5.

**Figure 9:**
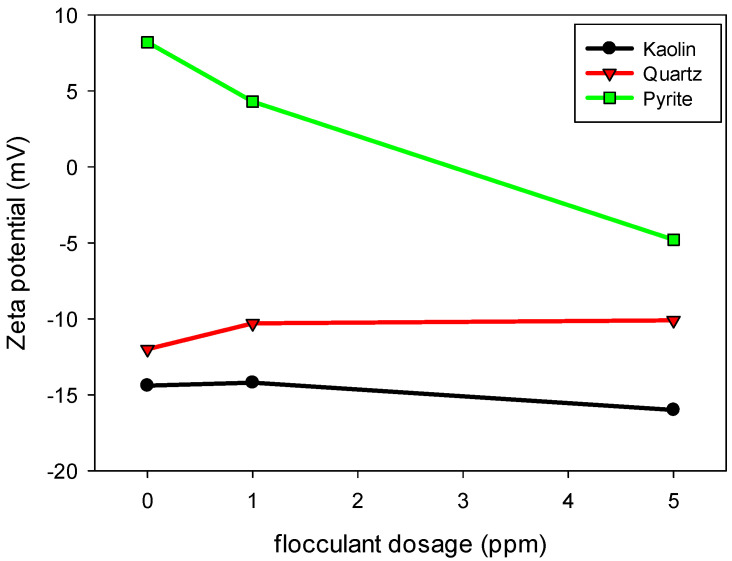
Zeta potential of kaolin, quartz and pyrite in the presence of industrial water and SNF 704 flocculant at pH 10.5.

Figure 8 shows the flocculant adsorption densities [g of flocculant/tonne of dry solid] on the surface of each mineral evaluated. The results confirm that pyrite exhibits the highest flocculant adsorption over the entire concentration range analyzed, with a progressive increase in the amount adsorbed up to approximately 320 g/t when the concentration of SNF 704 in the solution reaches 30 ppm. This behavior indicates a high affinity between pyrite and flocculant, which can be attributed to the surface charge of pyrite, which, in the presence of industrial water, acquires a cationic character (Figure 9), thereby favoring interaction with the anionic polymers of the flocculant.

Kaolin also exhibits increased flocculant adsorption with increasing SNF 704 concentrations, albeit with a more moderate slope than pyrite. The amount adsorbed by kaolin reaches approximately 245 g/t at 30 ppm of flocculant, suggesting a lower affinity between the flocculant and the clay than that of pyrite.

In contrast, quartz shows considerably lower adsorption than the other minerals. The amount adsorbed by quartz reaches a maximum of 60 g/t at intermediate concentrations of SNF 704. Still, it tends to stabilize and decrease slightly at higher concentrations, which could indicate a saturation of the active adsorption sites on its surface.

These results demonstrate the differential affinity of the SNF 704 flocculant for the various minerals present in the suspension, explaining the behaviors previously observed in terms of sedimentation rate and floc formation. The high affinity of the flocculant for pyrite suggests that the polymer adopts a train-type conformation on its surface, favoring rapid flocculation and the formation of agglomerates, although with an early saturation of the active sites. This phenomenon has been analyzed in molecular dynamics studies by Quezada et al. [10], who demonstrated that, on surfaces with high affinity for anionic polymers, such as brucite in contact with HPAM, the proportion of train-type conformations increases, reducing the presence of loops that contribute significantly to the formation of polymeric bridges and, therefore, to the efficiency of the flocculant. Thus, flocculant dosage optimization should consider the mineralogical composition of the suspension to maximize thickening efficiency, explicitly incorporating the pyrite content in the process evaluation.

Additionally, differences in flocculant adsorption modes can be attributed to the electrostatic nature of mineral surfaces and their interactions with the polymer, which exhibits an anionic character under high alkalinity conditions. This aspect is analyzed in more depth in Section 3.6 through zeta potential measurements. This differential affinity has practical implications for process optimization; excessive flocculant dosage in pyrite-rich systems may result in inefficient usage and diminished returns in terms of floc growth or sedimentation performance.

### 3.6. Zeta Potential

Figure 9 illustrates the variation in the zeta potential of kaolin, quartz and pyrite particles with increasing SNF 704 flocculant dosage at a pH of 10.5. In the absence of flocculant, pyrite exhibited a positive zeta potential of approximately 8.2 mV, while kaolin and quartz exhibited negative values of −14.4 mV and −12.0 mV, respectively. These values reflect the differences in surface charge properties of the minerals under alkaline conditions. With increasing flocculant dosage, distinct behaviors were observed among the minerals, reflecting the combined effects of surface charge, cation adsorption and interactions with the anionic polymer:

- Pyrite: The zeta potential of pyrite decreased significantly, from 8.2 mV to approximately −5 mV, with the addition of 5 ppm of flocculant. This change indicates a strong electrostatic interaction between the anionic polymer and the positively charged surface of pyrite at pH 10.5. The adsorption of the flocculant induces a progressive neutralization of the positive charges on the mineral surface, allowing the polymer chains to extend and form bridges between particles, thus promoting flocculation.
  The marked reduction in zeta potential also suggests that the flocculant can partially neutralize the surface charges of pyrite, decreasing the electrostatic repulsions between particles and favoring their aggregation. It is essential to consider that the positive zeta potential of pyrite in industrial water is due to the presence of calcium and sodium cations, which form complexes that adsorb on the mineral surface. For reference, in distilled water at pH 10.5, the zeta potential of pyrite is −3.5 mV, indicating that the industrial environment significantly modulates its electrokinetic behavior;
- Kaolin: Unlike quartz, kaolin showed a progressive decrease in its zeta potential, becoming more negative as the flocculant dosage increased. This behavior suggests a moderate interaction between the flocculant and the kaolin surface, possibly mediated by ionic bridges with the salts dissolved in the solution. In addition, kaolin possesses a moderate cation exchange capacity due to the presence of negatively charged sites on its edges and basal surfaces, which can adsorb exchangeable cations such as Na^+^ and Ca^2+^ from the medium. The adsorption of anionic polymer chains may displace some of these cations, altering the structure of the electrical double layer and contributing to the increased negative surface potential. Therefore, the higher negativity of the zeta potential could be attributed to both the adsorption of polymeric chains and the dynamic exchange of surface cations, exposing more negatively charged functional groups toward the liquid phase and thereby increasing the overall negative charge of the system;
- Quartz: In the case of quartz, the zeta potential remained practically unchanged with increasing flocculant dosage, indicating minimal interaction between the polymer and the mineral surface. This behavior can be attributed to quartz’s low surface charge density and the limited availability of active sites for flocculant adsorption. The fraction of polymer that adsorbs on the quartz surface appears to be mediated by the presence of ions in the solution, which facilitates a weak interaction between the flocculant and the mineral substrate.

These surface charge dynamics directly influence the adsorption mode of the polymer, which in turn affects floc structure and performance. Therefore, controlling ionic composition and understanding zeta potential responses are essential for designing effective flocculation strategies.

## 4. Conclusions

This study evaluated the impact of pyrite on the flocculation and sedimentation efficiency of clayey tailings at pH 10.5, providing key information to optimize thickening in the presence of this mineral. It was confirmed that pyrite significantly reduces the sedimentation rate, despite its larger size and specific gravity, due to forming less compact and more porous flocs. The high affinity of the SNF 704 flocculant for pyrite, attributed to the electrostatic interaction between its cationic surface and the carboxylate groups of the polymer, generates preferential adsorption with a “trains” conformation, limiting the formation of effective polymeric bridges. Increasing the flocculant dosage improves the capture of fine particles; however, the flocculant efficiency is affected in systems with high pyrite content due to the rapid saturation of the adsorption sites. For example, at 30 g/t, sedimentation velocity decreased from 11.1 m/h to 6.0 m/h as pyrite content increased. At low dosages (10 g/t), a higher pyrite content favors the formation of larger flocs, while at higher dosages (30 g/t), the aggregates are smaller and less compact. This is consistent with the decrease in fractal dimension (from ~2.7 to ~2.4), reflecting more open structures that hinder consolidation. The result is consistent with the high affinity of pyrite with the flocculant, as this generates rapid floc growth. Still, when the flocculant dosage is very high, the surface becomes saturated, limiting growth by the conformation of trains. Furthermore, the fractal dimension of the aggregates decreased with increasing pyrite, indicating more open and porous structures.

These findings demonstrate that flocculation efficiency is not solely governed by particle size or mineral density, but rather strongly depends on the structure of the aggregates formed and the interaction mode between the flocculant and mineral surfaces. Optimizing flocculant dosage in relation to pyrite content is critical to ensure efficient sedimentation and water recovery, particularly in systems operating at high pH and with seawater or recycled water.

## Figures and Tables

**Table 1:** Parameters used for all test pulps.

Parameter	Values
$d_{p}\;(\mu m)$	15.6 ± 0.7
$\rho_{solid}\;(kgm^{-3})$	2716.6 ± 50.3
$\rho_{liquid}\;(kgm^{-3})$	1000
$g\;(ms^{-2})$	9.81
$\mu_{liquid}\;(Nsm^{-2})$	0.001021
$\phi_{solid}$	0.039

## Data Availability

The original contributions presented in the study are included in the article and further inquiries can be directed to the corresponding author.
